# Supplementary material for: From sequence to enzyme mechanism using multi-label machine learning
Source: BMC Bioinformatics. 2014 May 19;15:150. doi: 10.1186/1471-2105-15-150 (PMC4229970; doi:10.1186/1471-2105-15-150)
Supplement: Additional file 2 — Java code of ml2db. Additional file ml2db_code.tar.gz contains the Java source code to run the multi-label machine learning experiments and save the results to database. The code’s Javadoc is included. [file 1471-2105-15-150-S2.zip › additional file 2/ml2db/ml2db/doc/mulan/dimensionalityReduction/LabelPowersetAttributeEvaluator.html]

LabelPowersetAttributeEvaluator


JavaScript is disabled on your browser.


- Overview
- Package
- Class
- Use
- Tree
- Deprecated
- Index
- Help

- Prev Class
- Next Class

- Frames
- No Frames

- All Classes

- Summary:
- Nested |
- Field |
- Constr |
- Method

- Detail:
- Field |
- Constr |
- Method


mulan.dimensionalityReduction

## Class LabelPowersetAttributeEvaluator

- java.lang.Object
- - weka.attributeSelection.ASEvaluation
  - - mulan.dimensionalityReduction.LabelPowersetAttributeEvaluator

- All Implemented Interfaces:
  :   java.io.Serializable, weka.attributeSelection.AttributeEvaluator, weka.core.CapabilitiesHandler, weka.core.RevisionHandler

  ---

    

  ```
  public class LabelPowersetAttributeEvaluator
  extends weka.attributeSelection.ASEvaluation
  implements weka.attributeSelection.AttributeEvaluator
  ```

  Performs attribute evaluation using the label powerset transformation. For
  more information, see

  Author:
  :   Grigorios Tsoumakas

  See Also:
  :   Serialized Form

- - ### Constructor Summary

    Constructors

    Constructor and Description

    `LabelPowersetAttributeEvaluator(weka.attributeSelection.ASEvaluation x,
    MultiLabelInstances mlData)`

    Constructor that uses an evaluator on a multi-label dataset
  - ### Method Summary

    Methods

    | Modifier and Type | Method and Description |
    | --- | --- |
    | `void` | `buildEvaluator(weka.core.Instances arg0)` Not supported |
    | `double` | `evaluateAttribute(int attribute)` Evaluates an attribute |

    - ### Methods inherited from class weka.attributeSelection.ASEvaluation

      `forName, getCapabilities, getRevision, makeCopies, postProcess, runEvaluator`
    - ### Methods inherited from class java.lang.Object

      `equals, getClass, hashCode, notify, notifyAll, toString, wait, wait, wait`

- - ### Constructor Detail


    - #### LabelPowersetAttributeEvaluator

      ```
      public LabelPowersetAttributeEvaluator(weka.attributeSelection.ASEvaluation x,
                                     MultiLabelInstances mlData)
      ```

      Constructor that uses an evaluator on a multi-label dataset

      Parameters:
      :   `x` -
      :   `mlData` -
  - ### Method Detail


    - #### evaluateAttribute

      ```
      public double evaluateAttribute(int attribute)
                               throws java.lang.Exception
      ```

      Evaluates an attribute

      **Specified by:**
      :   `evaluateAttribute` in interface `weka.attributeSelection.AttributeEvaluator`

      Parameters:
      :   `attribute` - the attribute index

      Returns:
      :   the evaluation

      Throws:
      :   `java.lang.Exception`


    - #### buildEvaluator

      ```
      public void buildEvaluator(weka.core.Instances arg0)
                          throws java.lang.Exception
      ```

      Not supported

      **Specified by:**
      :   `buildEvaluator` in class `weka.attributeSelection.ASEvaluation`

      Parameters:
      :   `arg0` -

      Throws:
      :   `java.lang.Exception`


- Overview
- Package
- Class
- Use
- Tree
- Deprecated
- Index
- Help

- Prev Class
- Next Class

- Frames
- No Frames

- All Classes

- Summary:
- Nested |
- Field |
- Constr |
- Method

- Detail:
- Field |
- Constr |
- Method
